# A Community-Based Assessment of Attitudes, Health Impacts and Protective Actions During the 24-Day Hangar Fire in Tustin, California

**DOI:** 10.3390/ijerph22071003

**Published:** 2025-06-26

**Authors:** Shahir Masri, Alana M. W. LeBrón, Annie Zhang, Lisa B. Jones, Oladele A. Ogunseitan, Jun Wu

**Affiliations:** 1 Department of Environmental and Occupational Health, Joe C. Wen School of Population & Public Health, University of California, Irvine, CA 92697, USA; anniez10@uci.edu (A.Z.); oladele.ogunseitan@uci.edu (O.A.O.); 2 Department of Health, Society, and Behavior, Joe C. Wen School of Population & Public Health, University of California, Irvine, CA 92617, USA; alebron@uci.edu; 3 Department of Chicano/Latino Studies, School of Social Sciences, University of California, Irvine, CA 92697, USA; 4 Institute for Clinical & Translational Science, The University of California, Irvine, CA 92617, USA; lbj@uci.edu; 5 Department of Population Health & Disease Prevention, Joe C. Wen School of Population & Public Health, University of California, Irvine, CA 92617, USA

**Keywords:** structure fire, survey, air pollution, Tustin hangar fire

## Abstract

Fire events can impact physical and mental health through smoke exposure, evacuation, property loss, and/or other environmental stressors. In this study, we developed community-driven, cross-sectional online surveys to assess public attitudes, health impacts, and protective actions of residents affected by the Tustin hangar fire that burned for 24 days in southern California. Results showed the most frequently reported fire-related exposure concerns (93%) to be asbestos and general air pollution and the most commonly reported mental health impacts to be anxiety (41%), physical fatigue (37%), headaches (33%), and stress (26%). Nose/sinus irritation was the most commonly reported (26.0%) respiratory symptom, while skin- and eye-related conditions were reported by 63.0% and 72.2% of the survey population, respectively. The most commonly reported health-protective actions taken by residents included staying indoors and/or closing doors and windows (67%), followed by wearing face masks (37%) and the indoor use of air purifiers (35%). A higher proportion of low-income residents had to spend money on remediation or other health-protective actions compared to high-income residents. Participants overwhelmingly reported disapproval of their city’s and/or government’s response to the fire disaster. Findings from this study underscore the potential impacts of major pollution events on neighboring communities and offer critical insights to better position government agencies to respond during future disasters while effectively communicating with the public and addressing community needs.

## 1. Introduction

Fire events can impact health through direct thermal injuries but also by causing mental health impacts from evacuation, property loss, and/or other environmental stressors. Additionally, fire-related smoke can worsen air quality, causing elevated exposures to fine particulate matter, gases, and volatile organic compounds that are associated with both respiratory and cardiovascular disease [1,2,3,4,5,6]. Evidence also links wildfire smoke exposure with increased emergency room visits for asthma and other conditions [6] and maternal exposure (during late pregnancy) with reduced birth weight and preterm birth [7].

While extensive research exists on the influence of wildfires on ambient air quality, the impacts of localized structure fires are often harder to characterize due to their smaller scale (and plume size), variable composition of source fuel, and shorter burn duration, which renders exposure and health impacts harder to detect. Limited air pollution measurements collected by firefighters and researchers nonetheless show elevated concentrations of crustal elements (e.g., aluminum, calcium, iron, and zinc) along with antimony, lead, chromium, copper, and various VOCs during structure fires [8,9,10]. During the 2018 California Camp Fire, which burned 18,804 structures [11] (e.g., residential, community- and faith-based, business, and school), elevated copper and zinc levels were similarly detected by the Point Reyes monitoring station, as well as lead concentrations that were 40 times over baseline [5]. While studies are not yet published, the 2025 Palisades and Eaton fires in Los Angeles, California, which burned over 16,000 structures combined [11], are likely to have left a similar air quality fingerprint.

In London, contamination following the Grenfell Tower (housing complex) fire demonstrated soil concentrations of dibenzo-p-dioxin, benzene, and polycyclic aromatic hydrocarbons to be 60 to 160 times above baseline [12], which was similar to the signature of contaminants found in the wake of the World Trade Center (large office complex) collapse in New York City in 2001 [13,14]. In the latter catastrophe, asbestos, polychlorinated biphenyls, and polychlorinated dibenzofurans were also reported. The variation in pollutants detected in the aftermath of such fires can be attributed to differences in structure composition, including their contents and building materials.

In addition to physical harm caused by chemical exposures, including the exacerbation of asthma, emphysema, and other chronic respiratory diseases, research has shown higher rates of mental health conditions such as post-traumatic stress disorder (PTSD), depression, stress, and anxiety following major fires. In a meta-analysis of 62 peer-reviewed studies, To et al. (2021) showed the consistent development of symptoms of depressive disorders and PTSD among adults following wildfire catastrophes, with prevalence rates three months post-fire of up to 60% and which persisted up to 10 years post-fire [15]. Similarly, rates of PTSD and depressive disorders were very high in children and adolescents post-fire, with anxiety disorders also elevated [15]. The risk of developing such mental health disorders after a fire can be influenced by witnessing homes burning down, fearing personal or familial injury or death, loss of loved ones to the fire, experiencing property damage, or feeling inadequate support from loved ones and the government [15]. Among the pediatric population, fear of losing their parents was a key predictor of increased emotional distress post-fire, even more so than fear for their own life [15]. If prolonged, mental stress can lead to the development of substance abuse, which has been observed among fire-impacted populations [15]. More broadly, prolonged depression has been linked with increased rates of self-harm and lower life expectancy [16,17].

Beyond direct injuries or the loss of life or property, factors causing fire-related mental distress may include solastalgia (the mourning of alterations in one’s natural environment), fear, school closures (leading to social isolation and hindered academic performance [18]), and road closures (causing traffic that impedes emergency responders and residents’ ability to commute to work [19]). In one study, feelings of unpreparedness and lack of community action plans were related to feelings of individualism, no control, and a lack of trust [20]. In general, proper mitigation of fires and adequate support from the community and the government may reduce the risk of individuals developing PTSD and depression following a fire [15].

Prior studies on the health impacts of fire-afflicted communities are few and typically face limitations that weaken the generalizability of their results. Of those that exist, impacts are often examined through a single lens (e.g., mental health), thus not capturing the full range of potential impacts suffered by community members. Similarly, such studies are seldom community-driven (involving community participation in the drafting of survey design) and often do not assess community perceptions of government disaster response, therefore missing an opportunity to provide feedback to improve future actions and intervention strategies.

Another challenge facing survey-based studies of communities is the threat posed by web robot participants (or “bot attacks”) during online survey data collection. Bots can be highly sophisticated and run automated tasks (scripts) with the intent of imitating human activity and earning participation dollars. Such attacks pose an obstacle for researchers and other entities seeking to evaluate insights and perspectives among the public, particularly amongst populations who may be highly mobile or have limited communication tools due to risk or damage to their homes or community and who thus may be less likely to be reached via in-person, mail, or phone-based survey approaches. Though tools can be deployed to help researchers identify and screen for inauthentic bot participation, bots have grown more sophisticated over time and can overcome many screening practices and tools.

One population of interest as it relates to post-disaster impacts is that which surrounded the North Hangar in the city of Tustin, California, during a major fire in 2023. In this study, we surveyed a demographically diverse sample of residents surrounding the Tustin hangars in order to evaluate the public impacts and perspectives regarding this major fire event and the related government response. Specifically, we addressed the following key questions: (1) What was the prevalence of health outcomes, and did such impacts vary by sociodemographic characteristics? (2) Which health-protective behaviors did residents adopt during the fire, and was residents’ willingness to engage in (and awareness of) such actions correlated with sociodemographic characteristics? (3) Did residents consider the government response adequate? (4) How was hazard communication disseminated to residents, and was it considered adequate during the fire? We also examined the prevalence of bot infiltration and the effectiveness of our automated bot screening procedures.

Based on our review of the literature and previous research and engagement with the impacted communities, we hypothesized that respiratory health outcomes would account for the majority of health issues reported by residents, that higher socioeconomic status would be associated with more health-protective actions taken by residents, and that community responses to government actions would be generally unfavorable. Separately, based on our previous survey work, we anticipated a high degree of bot infiltration but anticipated our automated screening techniques to be adequate to remove inauthentic survey responses.

## 2. Methods

### 2.1. Tustin Hanger Fire Incident

The city of Tustin is home to approximately 27,000 households and 80,000 residents, 6.2% of whom are under age 5 and 13.1% elderly (age 65+) [21]. In addition to being a residential hub, Tustin also houses two large marine air stations that were built during wartime in 1942. Nearly 28,000 m^2^ and 17 stories high, each structure is among the largest wooden structures ever built. On 7 November 2023, the North Hangar caught fire and burned for 24 days. Emergency response personnel from the Orange County Fire Authority (OCFA) were initially mobilized to extinguish the fire but were unable to do so due to the size of the wooden structure. Given such limitations, combined with safety concerns for the fire fighters, the OCFA decided to allow the fire to burn while applying fire suppression to limit its spread. Though the hangers, when initially built, were not adjacent to homes and businesses, today, the region has become a highly developed urban area, with each hanger sitting within just 200 m of numerous residential and commercial blocks. As such, the fire presented a major public health threat and community concern.

During the fire, emergency relief efforts were complicated by multiple layers of government jurisdiction, including the city, county, state, and federal level, along with the ownership of the structure itself by the U.S. Navy. This led to decisions that were widely varying and, in some cases, inconsistent with one another, in turn causing confusion and frustration among the public. An example is Orange County having declared a state of emergency [22] while California Governor Gavin Newsom refused to declare such an emergency [23]. The Governor’s decision also ran counter to efforts by local members of congress to issue an emergency proclamation [24] and was considered contentious in part as it eliminated the state’s eligibility for certain funds that may have been granted to support residents’ temporary evacuation and relocation.

On 8 November (one day into the incident), the South Coast Air Quality Management District (SCAQMD) published a press release and environmental monitoring report that confirmed the presence of asbestos fibers (up to 37% by mass) in fire debris found on the ground east of the North Hangar [25,26], followed by subsequent reports that showed no detectable asbestos and no abnormal VOC concentrations in air samples [27]. Subsequent air monitoring for 16 metals (e.g., lead, arsenic, cadmium, chromium, etc.) was also carried out and published daily throughout the course of the fire, confirming short periods of “elevated levels of lead and arsenic inside the area of the smoke plume” [27]. Once published, such results were quickly circulated over the news, allaying fears about certain exposures while stoking fear about others.

While the exact cause of the Tustin hanger fire remains unknown, a recent investigation by LAist suggested its potential connection with a break-in at the facility a day prior (by those looking to steal copper wire) [28]. To date, the U.S. Navy has pledged USD 88 million to aid in remediation costs, which is an amount commensurate with what the city had spent as of the pledge [29,30].

### 2.2. Study Design, Recruitment, and Sampling

A community-driven, cross-sectional online survey was developed to assess public attitudes and health impacts during the Tustin hangar fire. In conjunction with addressing research aims, questions were designed to assess community needs following a virtual public townhall meeting hosted by the Orange County Health Care Agency on 28 December 2023. In addition, we participated in community-information forums for impacted communities, particularly the Columbus Square neighborhood during the immediate aftermath of the fire in November, 2023. Specifically, the survey sought to assess three separate domains: (1) health and economic impacts; (2) health-protective measures taken by Orange County residents; and (3) public perceptions of government response and information dissemination during and after the active fire incident, which we anticipated to be interrelated as depicted in our conceptual framework (Figure S1).

**Figure 1:**
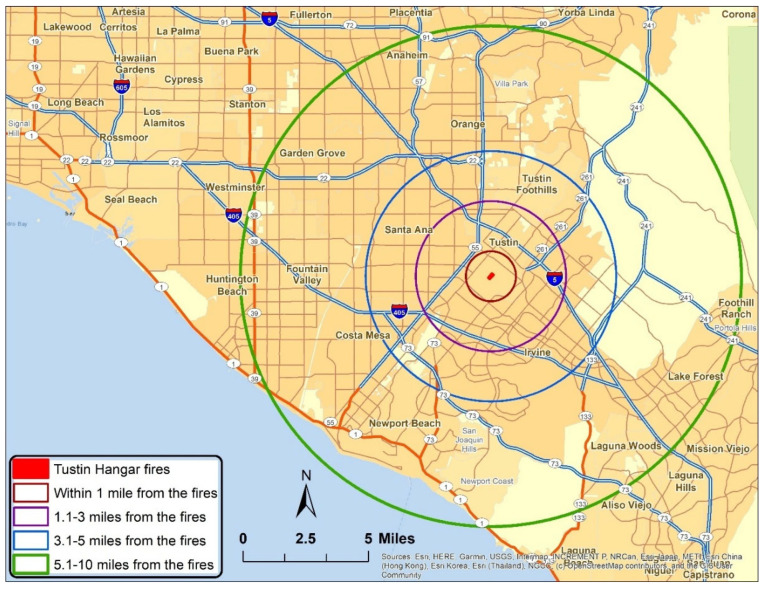
Map showing Tustin hangar fire and surrounding cities and buffer distances investigated in survey.

Two questionnaires were developed to solicit input from community residents and local school teachers, respectively. The teacher survey included all questions from the community survey in addition to questions evaluating the unique perceptions of school-wide responses and student-related activities. Figure 1 shows the location of the hangar fire and surrounding communities. Given its proximity to the ocean, the region is influenced by a land–sea breeze that reverses on a diurnal basis, likely resulting in two primary plume trajectories and a greater denominator of affected residents. Though it is possible that residents who responded to this survey tended to be those most affected (residing under the dominant plume paths), it was beyond the scope of the present study to examine spatiotemporal data of meteorological parameters. Prior to dissemination, surveys were approved by the Institutional Review Board (IRB) of the University of California, Irvine.

Both surveys were administered during March 2024. Data were collected and managed using Research Electronic Data Capture electronic data capture (REDCap) tools hosted at the University of California, Irvine [31,32]. REDCap is a secure, web-based software platform designed to support data capture for research studies, providing (1) an intuitive interface for validated data capture; (2) audit trails for tracking data manipulation and export procedures; (3) automated export procedures for seamless data downloads to common statistical packages; and (4) procedures for data integration and interoperability with external sources. Survey participant recruitment methods included snowball sampling through local community groups (e.g., university contacts, non-profit organizations, and social networks) who circulated the survey in English and Spanish via email listservs and social media platforms (i.e., Facebook and Instagram).

The intent of the survey was to identify community needs regarding the Tustin hangar fire and to enable the reporting of information to governmental agencies at various levels (e.g., cities, county, navy base, regional, state, and federal) that were involved in fire response efforts and/or whose residents and workers were affected by the incident. The study results have the potential for translation into strategies and policies designed to improve emergency response procedures, and to reduce negative health outcomes experienced by residents. Additionally, responses to survey questions can provide a baseline assessment of health outcomes related to exposure experiences during the Tustin hangar fire.

### 2.3. Data Cleaning and Removal of Automated Bot Responses

A total of 1385 community and 37 teachers-only questionnaires were completed. However, only 54 surveys were selected for further analysis because we rejected automated responses completed by online software applications known as web robots, or “bots” [23]. Bots can be highly sophisticated and run automated tasks (scripts) with the intent of imitating human activity and earning reward money [33]. Though it cannot be confirmed, the extensive bot attack that infiltrated this survey may be related to the promise of USD 10 of compensation awarded to randomly selected participants, which was noted in the online advertisement distributed to potential survey respondents.

Exclusion criteria for completed questionnaires included responses that fit patterns of bot participation, such as commencement of numerous questionnaires at the exact same time (as indicated by survey timestamps), which was atypical compared to the far less frequent background rate of completed survey entries, as well as by noticing a pattern of similar contact emails (e.g., first name, last name, X numbers). In addition to developing criteria-based screening, this led our team to also develop and apply the following automated and manual bot screening processes to clean the collected survey data, ultimately leading to the identification and removal of 1368 (96.0%) observations that were considered to be “bots”:

1. Criteria-Based Screening: 23 respondents were eliminated due to being either below the age threshold (18 years old) or not residing in California.
2. Automated Bot Screening Process: To detect bot responses, several layers of automated screening were established, including the removal of surveys with early completion times (<3 min, corresponding to ~10th percentile completion time), start and completion times that were identical to one another, and discrepancies between residential address and reported distance from fire. This process resulted in the elimination of 1020 additional observations.
3. Manual Bot Screening Process: When completing the surveys, each participant was asked to provide their phone contact number. Of the 342 community observations that remained following the two previously described data cleaning processes, 299 (87%) contained phone numbers, each of which was dialed as a final step to manually confirm authentic participation in the survey (by speaking with the participant). (We dialed up to two additional times if a voicemail was reached.) Among this group, 50 (16.7%) participants verbally confirmed their participation in the survey, while those removed from the analysis included 153 (51.2%) participants whose phone numbers led to inactive lines (e.g., no dial tone), 61 (20.4%) whose phone numbers led to business lines (e.g., dental office, nail salon, etc.) where the participants did not appear to be employed, while 32 (10.7%) led to participants who confirmed they did not actually participate in the survey, and 3 (1%) led to those who could not remember if they participated.

Given the small sample size, all participants of the teachers-only survey were subjected to the Step 3 bot screening procedure, and so Step 2 was skipped. This resulted in 4 (11.4%) verbally validated surveys and 33 (89.2%) being excluded due to phone numbers leading to business (65.7%) or inactive lines (20.0%), individuals confirming they did not participate (2.9%), and missing phone numbers (5.4%).

Since participants who did not provide a phone number (N = 38 following Step 3 cleaning) could be authentic human participants (who perhaps did not feel comfortable leaving their contact info), such responses were examined separately to compare summary statistics with those of validated responses. The data showed drastic differences in demographic characteristics and responses to key questions between this group and the verified group (e.g., 50% reporting to have visited a doctor due to the hangar fire, compared to just 12% in the verified population), which affirmed our decision to exclude these observations from the main analysis.

### 2.4. Measures and Analysis

All participants were asked to report the proximities of their residence and workplace to the Tustin hangar fire according to six options (<1 mile, 1–3 miles, 3–5 miles, 5–10 miles, and >10 miles), with proximities greater than 10 miles considered less impacted (as affirmed by the absence of community-reported fire debris beyond this distance [34]). Demographic indicators assessed were gender, age, race/ethnicity, educational attainment, and household income.

To assess Domain #1 of our survey, health/medical-related measures included “yes/no” questions about participants’ health status during or immediately after (within 2 weeks of) the fire (e.g., *Did you see a doctor, nurse or other health care professional due to sickness related to the Tustin hangar fire?*). Similar “yes/no” questions were asked regarding access to health-protective interventions and experiencing financial impacts, fire-related property contamination (e.g., fire ash), and missed school or workdays due to the fire.

As a measure of worry, participants were asked to rank the extent to which they agreed with six statements (1 = strongly disagree, 2 = disagree, 3 = neutral, 4 = agree, and 5 = strongly agree) (e.g., *I worried about my personal health risks and that of my family members caused by the Tustin hangar fire*). Additionally, a series of “check all that apply” options were provided in response to numerous questions regarding the types of illnesses participants acquired during or immediately after the fire, consistent with other health-related questionnaires, including the U.S. Centers for Disease Control and Prevention’s Behavioral Risk Factor Surveillance System (BRFSS) and California Health Interview Survey (CHIS) [35,36]. Illnesses selected for inclusion were those which are known to result from exposure to air pollution and disaster events, including mental health impacts and impacts to the eyes and skin and respiratory and cardiovascular systems.

Regarding Domain #2, the survey included “check all that apply” questions about health-protective measures taken by residents, including their potential use of indoor air purifiers, closing doors and windows, cleaning surface dust, etc. Similarly, a question was included evaluating whether residents had access to certain interventions (e.g., *Do you own an air purifier?*).

Domain #3 measures evaluated participants’ agreement with statements about the government response and dissemination of health-protective information during the fire (i.e., *The city did a good job reaching out to affected community members during the Tustin hangar fire as it relates to safety*). Finally, a series of “check all that apply” options were provided in response to numerous questions regarding the hazard communication methods that were considered most effective by residents. Following the administration of the online survey, all survey responses were exported as a .csv file and subsequently analyzed using SAS software, Version 9.4 [37].

When evaluating whether percentages differed according to race/ethnicity and income levels, statistical significance was evaluated at the *p* = 0.05 level using the chi-squared statistic.

## 3. Results

### 3.1. Sociodemographic Characteristics of Respondents

Table 1 presents the socioeconomic and demographic characteristics of respondents. Within validated survey responses (N = 54), a slight majority were female (53.7%). Among participants, 61% were between ages 25 and 44, with roughly one-third below this age range. A majority identified as Latina/o/x/é (48.2%), followed by White/Caucasian (35.2%) and Asian American/Asian (11.1%). About 45% reported a household income between USD 50,000 and USD 100,000, with 24% reporting income below this range. Approximately two-thirds reported having a bachelor’s degree or higher, with 15% having some college experience and the remainder having junior college experience or less. All participants resided in Orange County (OC), California, with the majority located in Santa Ana (31.5%), Irvine (20.4%), and Tustin (18.5%).

### 3.2. Proximity of Respondents to Hangar Fire

**Figure 2:**
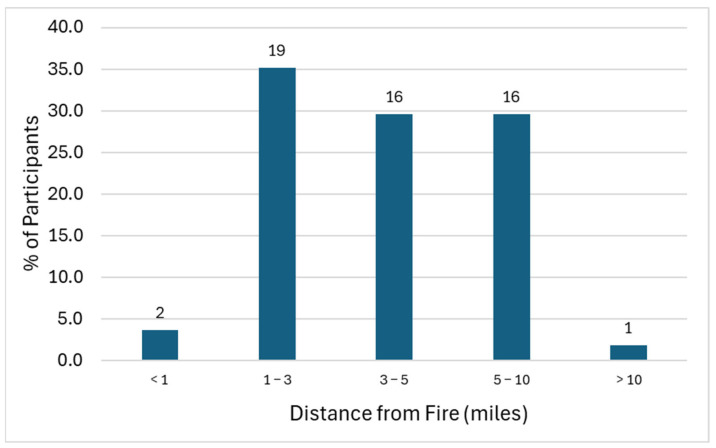
Geographic proximity of survey participants to Tustin hangar fire expressed as percentages (*y*-axis) and number of people (values above each bar).

All survey participants except for one reported living within 10 miles of the hangar fire, with 69% residing within 5 miles of the fire and ~4% (n = 2) within just 1 mile from the fire (Figure 2). A total of 76% of participants reported working within 10 miles of the hangar fire, with 54% and 8% working within 5 miles and 1 mile from the fire, respectively (18% worked >10 miles from the fire, and 6% did not work). Forty-one participants (82%) were sufficiently close to the fire to report seeing or smelling smoke related to the Tustin hangar fire, with 54% noticing dust or ash settling around their property or neighborhood.

### 3.3. Impacts of the Hangar Fire on Health and Economic Situation of Respondents

Of the survey participants, five (9%) reported to have a child or other family member who had to see a doctor or other health care professional due to sickness related to the Tustin hangar, one of whom had to also personally receive such attention due to sickness.

The most commonly reported mental health impact related to the Tustin hangar fire was anxiety (41%), followed by physical fatigue (37%), headaches (33%), stress (26%), and fear, depression, and trouble sleeping (19%, respectively). Similarly, 19% of participants reported having no mental health impacts related to the fire. The percentage of “no impact” responses dropped by half (9.5%) when examining only those residing within 5 miles of the fire.

Nose/sinus irritation was the most commonly reported (26.0%) respiratory symptom to occur during or immediately after the Tustin hangar fire. Coughing, general difficulty breathing or shortness of breath, sneezing or a runny/blocked nasal passage, and a sore/irritated throat were each reported by approximately 20% of participants, while wheezing or whistling in the chest was reported by 13.0%. Asthma attacks were reported by 7.4% of participants (including four participants whose attack required a nebulizer and two that required an inhaler), while other symptoms, including contraction of a cold and bronchitis, were reported by less than 5% of participants. Relatedly, the exacerbation of angina symptoms was reported by six participants (7.4%) during the study period. Just 13 participants (24.1%) reported no respiratory or cardiovascular health impacts, while 28% reported three or more such symptoms. These statistics can be viewed graphically in Figure S2 of Supplemental Materials.

Skin- and eye-related conditions were reported by 63.0% and 72.2% of the survey population, respectively, with dry and itchy skin being the most commonly reported skin-related symptom (31.5%), followed by itch/scaly rashes and acne (16.7%, respectively), and itchy or watery eyes (33.3%) being the most commonly reported eye-related symptoms. These statistics can be viewed graphically in Figure S3.

In terms of economic impacts, 18.5% of participants had to spend money on clean-up or other strategies to reduce health risks due to the fire. This percentage was higher (23.1%) for the lower-income half of the survey population compared to the higher-income half (14.3%). Additionally, 9.3% (n = 5) reported having to take time off work due to the Tustin hangar fire, and 27% of those with school-aged children (n = 10) reported at least one child missing school due to the fire. Although not statistically significant, a higher percentage of lower-income participants (11.5%) reported having to miss work due to the fires compared to their higher-income counterparts (7.5%). While home ownership was not evaluated, 39% of participants reported concern over their own or their neighborhood’s housing values due to the fire.

### 3.4. Expression of Concerns and Actions Taken by Survey Respondents

When asking participants to identify their concerns about materials or potential toxicants resulting from the Tustin hangar fire, the most common concerns (93%) were asbestos and general air pollution (e.g., particulate matter, VOCs, etc.). Lead and other heavy metals were a concern identified by 67% and 56% of participants, respectively, with zero participants reporting no concerns about exposures from the fire.

Overall, 83% of respondents reported a sense of worry about personal or familial health risks related to the fire, with 57% reporting such worry about their pets. Similarly, roughly three-fourths of participants worried about where they could safely spend time in their community and/or the health risks of time spent in their community due to the fire, while 69% (of those with children) worried about the fire-related health risks of sending their children to school.

When considering the actions that participants took to protect their health or the health of their housemates during or after the Tustin hangar fire, the most commonly reported action was to stay indoors and/or close doors and windows (67%), followed by wearing face masks (37%), indoor use of air purifiers (35%), cleaning dust around the home (30%), removal of shoes before entering the home and/or taking long showers (22%), and evacuation (13%). There were only 10 participants (19%) who reported taking just one of these actions, with the majority (67%) taking between two and four of these actions.

Despite less than one-third of participants reporting to have used an air purifier as a protective action, just 46% of participants reported owning an air purifier that could be used in the household, with 50% reporting that they did not own an air purifier and the remainder unsure. The proportion of those owning an air purifier was notably lower (31%) among lower-income participants (household income < USD 50,000) compared to higher-income (≥USD 100,000) participants (56%), although this difference did not reach the level of statistical significance. Of those who reported using an air purifier, 37% reported that they did not close their doors or windows as a protective action (although it is possible that doors/windows were closed by coincidence). As a hypothetical, participants were asked whether they would evacuate their home or residence if recommended by emergency officials, with 74% responding in the affirmative, 15% stating they would not evacuate, and the remainder unsure.

### 3.5. Perceptions of Governmental Response and Outreach Among Respondents

When asked about the government’s effectiveness (referring broadly to any local or non-local government entity) reaching out to affected communities during the Tustin hangar fire, residents overwhelmingly reported disapproval about their city’s and/or government’s response, with about 2 to 5 times more residents expressing disapproval compared to approval on five different metrics. Specifically, 20.4% of respondents felt the city and/or county did a good job, compared to 53.7% who felt they did not do a good job and the remainder who felt neutral or unsure. Similarly, 16.7% of participants felt the government provided enough information to understand the potential health risks caused by the Tustin hangar fire, compared to 64.8% who felt the opposite. Also, 13.0% of participants felt governmental messaging was clear and effective compared to 63.0% who felt the opposite (the remainder either neutral or unsure).

Four participants (7.4%) reported feeling that sufficient measures had been taken by the government to mitigate the health risks caused by the Tustin hangar fire compared to 55.6% who felt insufficient measures had been taken (the remainder felt either neutral or unsure). During the Tustin hangar fire, the “debris reporting portal” was established for residents to report fire-related debris during or after the fire. However, less than a quarter of respondents (22.2%) knew about this resource. In general, only 29.6% of community members felt they knew about reliable information sources to stay updated on fire-related news, with 50.0% feeling they did not have reliable sources.

**Figure 3:**
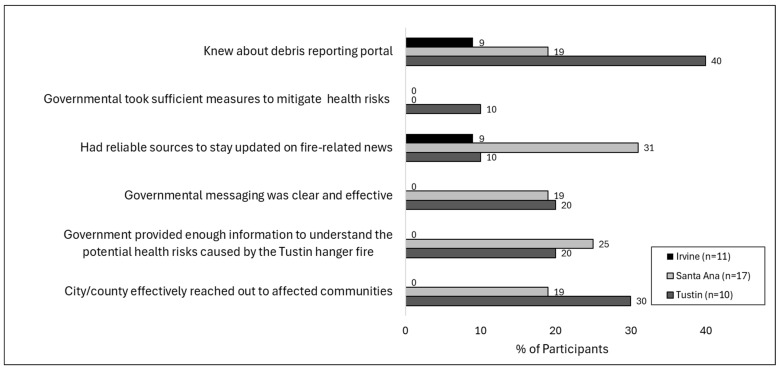
Residents’ perception of government response to Tustin hangar fire, by city.

As shown in Figure 3, perceptions of satisfaction with government responses and community access and awareness of relevant information varied greatly across the three main cities where participants resided. Perceptions were most favorable in Tustin and Santa Ana compared to Irvine. Specifically, on four questions evaluating whether government actions and community outreach were clear, sufficient, and comprehensive, none of the Irvine residents responded favorably.

**Figure 4:**
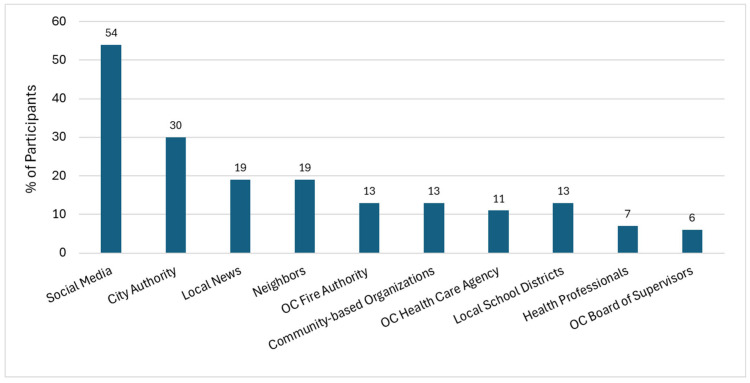
Information sources from which residents reported receiving city- or county-based warnings related to the Tustin hangar fire.

In obtaining warnings from the city or county during the fire period, Figure 4 shows that social media was the most commonly reported information pathway (54%), followed by residents’ city authority (29.6%), the local news (18.5%), neighbors (by 18.5%), and the Orange County Fire Authority (by 13.0%). Local schools or school districts, community-based organizations, the Orange County Board of Supervisors, the Orange County Health Care Agency, and health professionals were identified as sources by approximately 6 to 13% of participants.

Residents regarded an explanation of the associated health risks and underlying cause(s) of the fire to be the most helpful types of information the government could provide (87.0% and 83.3%, respectively), followed by a description of the type, location, and results of environmental testing (if any) carried out during/following the fire (69–76% of residents), the prevailing wind and debris patterns during and after the fire episode (72.2%), and the plan for post-fire cleanup of the Tustin hangar site and surrounding homes and/or park/greenspace areas (65–67%). The sharing of ways to participate in the tracking of health effects/concerns linked with the fire was identified by 61.1% of participants, and the post-fire cleanup plan for businesses was identified by half of participants.

**Figure 5:**
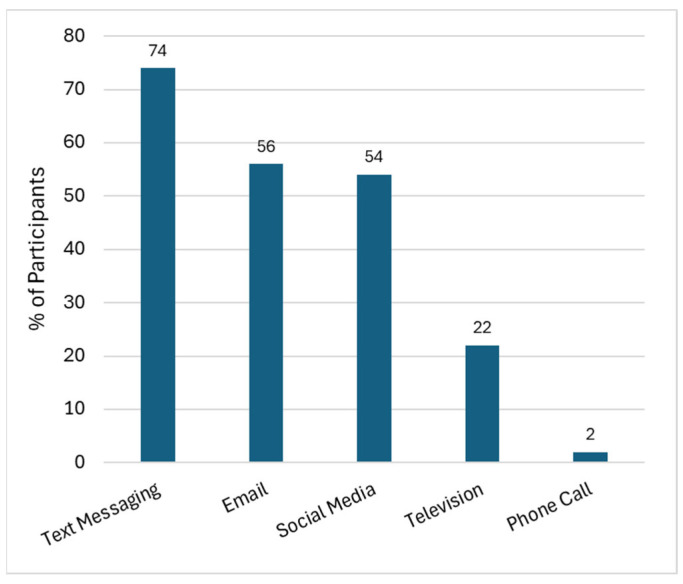
Proportion of residents identifying the “best” mode of hazard communication using check-all-that-apply format.

As shown in Figure 5, text messaging (74.1%) was overwhelmingly considered to be the best way to receive communications regarding hazards and health effects associated with the Tustin hangar fire, followed by email and social media (56% each) and televised messages (20.4%). Contact by phone calls was reported to be the best means of communication for only one participant (1.9%).

When asked whether participants wanted to receive a summary of the survey findings once completed and analyzed, 49 (90%) responded affirmatively. Moreover, 34 participants (63%) reported an interest in research opportunities related to the health effects of the Tustin hangar fire incident if available.

## 4. Discussion

This study presents an analysis of community perceptions and actions based on responses to survey questions disseminated to residents living within 10 miles of a major structure fire (Tustin hangar fire) that burned for over three weeks in southern California in 2023. Our research concerned three domains that consisted of health and economic impacts, health-protective measures adopted by residents, and public perceptions of government response and crisis communication and was guided by a conceptual framework that anticipated multidirectional interactions and the influence by socioeconomic factors. While we expected the hanger fire to impact health/economics, a potential two-way interaction existed between such impacts and residents’ perceptions of the government response (e.g., those experiencing such health impacts may have perceived a poor government response, while such perceptions may have also affected health). Similarly, governmental crisis communication and management may have directly affected the severity of the hanger fire and therefore created direct health/economic impacts. Yet it may have also had indirect impacts related to effective crisis communication (or lack thereof), including the dissemination of information that influenced residents’ adoption of health-protective actions. Meanwhile, socioeconomic factors may have affected residents’ ability to adopt health-protective measures (e.g., cost considerations) and/or their vulnerability to health/economic impacts (e.g., access to healthcare, existence of pre-existing health conditions, job flexibility during evacuation, etc.).

### 4.1. Survey Findings

#### 4.1.1. Health and Economic Impacts

Among residents, the majority reported seeing or smelling smoke related to the Tustin hangar fire (or noticing physical dust or ash settling around their property), thus confirming this study to have recruited participants who can be considered reasonably exposed to the fire event.

Mental health outcomes were widely reported among residents, including anxiety, fatigue, headaches, stress, fear, and depression. These outcomes may be related to the inadequate crisis communication and governmental response reported by community members, as discussed subsequently, as well as proximity to the hangar fire. Moreover, these findings are consistent with those previously reported in the wake of wildfires [15] and may suggest the persistence of longer-lived mental health outcomes such as depression, which should be examined in follow-up research.

Most participants reported experiencing respiratory or cardiovascular symptoms during or immediately after the fire, with nose/sinus irritation being the most commonly reported symptoms. While baseline conditions of sinusitis may account for some of this reporting, the symptoms reported in this survey were more than twice the national prevalence (11.2%) of sinusitis, suggesting that the hangar fire may have played an influential role [38].

Regarding current/active asthma, the prevalence in Orange County and the nation are approximately 8% [38,39], and the fraction of those with asthma who suffer from one or more asthma attacks per year is 39% [40]. Applying these statistics to the current survey population yields a highly conservative estimate of 3.1% of participants expected to have suffered an asthma attack during the five-week study period. By contrast, the prevalence was more than double in this sample, suggesting that air pollution resulting from the Tustin hangar fire may have exacerbated the occurrence of asthma attacks or triggered new asthma cases in the surrounding communities.

Dry and itchy skin, along with itchy/watery eyes, were also commonly reported (by at least one-third of participants). Seasonal allergies, which affect roughly a quarter of adults in the U.S. each year [41], are unlikely to explain the high reporting in this survey, particularly given that the hangar fire did not overlap with the allergy season. Eczema and food allergies, which affects roughly 7% of adults in the U.S. each year, may account for some of the skin-related outcomes reported in this study, though still falling short of explaining the entirety of skin conditions. Though other independent skin conditions may have played a role, fine particulate from the Tustin hangar fire may represent a contributing factor. Percutaneous absorption of air pollutants such as combustion-related PAHs can enter the skin through either trans-epidermal movement or absorption through hair follicles and sweat ducts. Upon entry, such exposures have been described as contributing factors for a number of skin disorders (including eczema) [42].

Lower-income participants, despite already being more economically vulnerable, were disproportionately represented among those who had to spend money on clean-up or other strategies to reduce health risks or to take time off work due to the fire. This finding underscores the ways in which issues of environmental injustice can arise in the wake of environmental disasters or persistent pollution—a pattern which has been widely documented in other settings [43,44,45,46,47].

#### 4.1.2. Community Concerns and Actions

Asbestos and general air pollution (e.g., particulate matter, VOCs, etc.) were of greatest exposure concern to residents, a finding that likely reflects early news and reports disseminated during the early phases of the fire, including the SCAQMD report that confirmed asbestos in surrounding fire debris, as described in Section 2.1 [25,26].

Similarly, participants’ concern over their neighborhood’s housing values due to the fire may have been influenced by County Assessor Claude Parrish comments that called the situation a “disaster” due to future home buyers likely avoiding the area (it is not yet clear how property values have been impacted) [48]. Following the fire, the regional tax assessor allegedly sent over 23,000 notices to surrounding homeowners regarding opportunities for property value reassessments, with properties suffering USD 10,000 in damage potentially eligible for a temporary value adjustment [48].

Fire-related health risks were also a concern to many parents who sent their children to school. In one case, a participant used the free-response section of the survey to express concern over the fact that schools in Tustin were closed due to the fire, while nearby schools in Irvine remained open. When paired with results reporting mental health impacts, this again affirms the potential for longer-term post-fire mental health impacts that should be investigated. Moreover, school closures themselves may produce mental health impacts, including increased anxiety and reduced learning, as was documented following the widespread school closures that accompanied the COVID-19 pandemic [49,50].

Many residents stayed indoors and/or closed their doors and windows to protect their health, which is a reassuring finding given the documented effectiveness of such measures in minimizing exposure to outdoor air pollutants [51]. Face masks were less common, yet still notably reported. Though face masks can be effective in minimizing air pollution, some masks (e.g., surgical masks) are far less effective than others (e.g., N95-rated) and therefore can provide a false sense of security against exposure to air pollutants. Similarly, improperly fitted facemasks and the presence of facial hair can drastically reduce their effectiveness. This survey did not ascertain the type of face masks used by residents nor whether they possessed facial air.

While indoor air purifiers can be highly effective at protecting against outdoor air pollutants, they were used only about as much as face masks. Unfortunately, however, half the survey population did not possess an air purifier and therefore could not use one even if desired. When examining those who owned an air purifier, the percentage was slightly higher among higher-income participants compared to those earning less, suggesting that the use of air purification may be related in part to economic resources. Importantly, among those who did own and use an air purifier, over two-thirds did not report the important step of closing their doors or windows as a protective action during the fire, thus potentially negating the benefits of using an air purification device. While doors/windows may have been closed by coincidence (not evaluated by survey), this nonetheless highlights a critical knowledge gap as it relates to the effective use of air purifiers and protection against outdoor air pollutants.

#### 4.1.3. Government Response and Outreach

Residents overwhelmingly reported disapproval about their city and/or other governmental agency’s response efforts, with Irvine residents expressing the greatest percentages of disapproval. The case of Irvine may be explained by a combination of genuine government failure regarding health and safety and/or a failure to relay relevant information to residents on the steps the government was taking during the incident. In the case of Santa Ana, however, the fact that zero residents felt their government took sufficient measures to mitigate health risks despite nearly one-third having reliable sources to stay updated on fire-related news suggests that residents’ disapproval of the government response was not likely due to lack of information but mostly a genuine failure on the part of the government to meet community needs and expectations regarding health, safety, and cleanup. That residents from Tustin systematically reported higher rates of government approval and information awareness may be reflective of a heightened mobilization by Tustin officials given that the fire incident and response efforts were centered in Tustin, while other cities such as Santa Ana and Irvine were also affected by the fires yet may have seen less local government mobilization.

Approval of the governmental responses during the Tustin hangar fire were similar to, though even lower than, that of state and national polling. According to the Public Policy Institute of California, for instance, only 29% percent of adults have a “great deal” of confidence in their government’s readiness to respond to wildfires [52]. Similarly, following the historic 2025 LA Fires, polling by the University of California Berkeley Institute of Governmental Studies found that 44% of Los Angeles residents felt their mayor—Karen Bass—did a “poor” or “very poor” job in responding to the fires [53]. A comparable lack of confidence and approval was observed following other crises, including the federal government’s COVID-19 response [54]. Compared to other nations, findings from prior research have shown the U.S. to rank relatively low in terms of public approval of the government’s COVID-19 response [55], perhaps reflecting the 20-year decline in the United States in the percent of Americans who trust their government to address domestic issues (currently at 39%) [56].

The factors influencing the public’s trust in government are likely complex and have been shown to correlate inversely with press freedom, through which critical information and public critiques of government can be widely shared [55]. However, major environmental health-related disasters throughout modern history, which have showcased critical failures in disaster preparedness and response, may also contribute to widely held skepticisms of government-related emergency response. For instance, during the infamous chemical factory explosion near Seveso, Italy, which exposed residents to high levels of dioxin, Zone A residents (nearest the site) were not evacuated until 2 weeks following the explosion, while Zone B residents were never asked to evacuate [57]. Ultimately, residents in both zones sustained adverse health effects [57]. Similarly, during the 1984 pesticide plant disaster in Bhopal, India (which killed thousands of residents within days due to methyl isocyanate exposure), and the infamous Chernobyl nuclear disaster of 1986, government and/or industry entities failed to provide a timely and adequate warning to downwind communities about the harmful emissions, thus leading to delayed evacuation and response efforts. Oil spills such as the 1989 Exon Valdez and 2010 Deepwater Horizon incidents have similarly been accompanied by poor disaster communication and response, leading to prolonged emissions to the environment and illnesses among first-responders, the public, and wildlife.

In the present analysis, low approval in government action appears reflected by the very low percent of participants who were aware of the debris reporting portal, which was established on November 14th (one week after fire began), for residents to report the presence of debris related to the fire. Additionally, most residents did not feel they possessed reliable sources to stay updated on fire-related news.

That over a quarter of participants reported that they were either unsure or would not evacuate if recommended to do so by emergency officials seems to further underscore a general sentiment of distrust among community members towards the government. Research by Kuligowski et al. (2022), however, highlights other factors that can reduce the tendency to evacuate, including lower risk perception, higher educational attainment, mid-range annual income (USD 50,000–USD 74,999), and more members residing in households [58]. Additionally, having pets was associated with lower risk perception, potentially due to a greater ability to cope or a sense of needing to be a source of protection for pets.

While showcasing popular disapproval of the government’s response to the Tustin Hanger fire, this study also provides valuable insights that enable the government to understand which of their activities were regarded as both favorable and unfavorable by the community. Social media, for instance, was the primary means through which most residents reported receiving warnings from the city/county about the Tustin hangar fire, suggesting that local governments may be adapting to shifts in news consumption pathways, since the large majority of U.S. adults (86%) now obtain their news from digital devices compared to radio, print publications, etc. [59].

Residents also identified various ways governments can succeed in crisis communication with residents; specifically, by providing information regarding the health risks and underlying cause(s) of the fire, along with information regarding environmental testing, cleanup, and prevailing wind patterns surrounding the fire. Residents also desired a way to track health effects/concerns linked with the fire, although no such portal has been provided.

Text messaging, email, and social media were each identified as the best ways to receive communications regarding hazards and health effects of the fire by half or more of respondents, whereas outreach through televised and phone communication were identified by one quarter or less of participants. The increased use of social media as a primary means of obtaining news (compared to more traditional news sources such as television and newspapers), especially among the younger demographic, is also consistent with findings from our prior work and others [59,60]. Social media can be an effective way to expeditiously disseminate emergency information during disaster situations and to therefore improve preparedness and minimize harm [61]. Similarly, social media can be an effective way for the public to gauge the sentiment of their community regarding an impending disaster. During Hurricane Ian, for instance, Karimiziarani et al. found “caution” to be one of the most common words discussed among residents using social media [61,62].

### 4.2. Community Engagement and Future Roadmap

Throughout the course of the Tustin hangar fire incident, community–government communication activities at the city level took the form of public town halls and city council meetings [63]. At the county level, the Orange County Healthcare Agency played an important role in responding to community inquiries and also organized/hosted a public webinar and hearing which included an expert on asbestos exposure and toxicity.

Despite such activities, the communication between residents and local governments was considered minimal and ineffective by many residents. This view was expressed by several vocal members of the audience during the community forum in which we participated during the aftermath of the main fire incident in November and December, 2024. In particular, residents of the Columbus Square neighborhood (which is closest to the hangar fire in Tustin) lamented inadequate communication from city and county officials about risks posed by toxic pollutants, support for evacuation, and potential risks associated with planned cleanup operations.

In Santa Ana, a neighboring city that was impacted by the fires as well as jurisdictional constraints in fire and community response, residents expressed their concerns via trusted community-based organizations, who, in turn, communicated resident concerns and frustrations to city and county officials. Unfortunately, residents’ concerns were not immediately heard or validated by city or county officials, which these officials in part attributed to other jurisdictions’ (e.g., other cities and federal agencies) responsibility for overseeing the fire response.

Moreover, the responses by different levels of government were not always consistent, as noted in Section 2.1, resulting in mixed messaging that has the potential to create confusion and to heighten anxiety and frustration among affected residents.

According to Sapiains et al. (2020), social participation is an essential element of fire prevention that is superior to top-down approaches, which can suffer from community distrust in government [20]. Additional insights from the prior literature suggest that the promotion of community participation and outreach during natural disasters can help build social capital and strengthen community resilience, hazard prevention, and address socioeconomic gaps relating to inequitable resource access [64,65]. Such efforts can potentially include a community-based mapping and/or record keeping effort to identify vulnerable households so as to construct an evacuation assistance plan, whereby disabled residents or those without cars can still reach safety (e.g., carpooling) in a timely manner. Moreover, the establishment of citizen action groups can be a valuable community asset to carry out these and other disaster-preparedness activities, including pre-disaster training workshops and drills, and also to facilitate emergency response and remediation efforts.

In terms of government response to a related type of disaster (wildfire impacts), in 2022, the U.S. Government Accountability Office (GAO) issued a report finding the federal approach to disaster recovery to be fragmented across more than 30 agencies and departments and at least 32 congressional committees [66]. The report recommended that agencies identify and take steps to better manage such fragmentation and that Congress consider creating an independent commission to identify reforms to the federal government’s approach to disaster recovery. To date, no such commission has been established.

### 4.3. Addressing Bots in Online Survey Data

Evidence from this survey demonstrated extensive infiltration by web-bots. This finding is disappointing yet consistent with prior research, including a recent study by Storozuk et al. (2020) that points out that 37.2% of internet traffic was attributable to bot activity in 2019 and that the proportion of bots designed to engage in criminal activity (relative to those designed to help users navigate the web) represented 24.1% of total bot traffic that year [67].

Several indicators of the presence of bot responses in our survey were the same as those observed by other researchers [67], namely, the concurrent influx of a high number of surveys on certain days or hours of the day without reasonable explanation, high survey completion speeds, the commencement and/or completion of many surveys at the exact same time, similar email patterns, and the completion of the survey despite a failure to meet eligibility requirements. Unlike prior researchers, however, bot responses to open-ended questions in our survey were not nonsensical, thus preventing us from using this as a tool to distinguish bots from humans [67]. This suggests that bots have improved in sophistication, allowing for more complex free-response answers that are more capable of mimicking humans.

While the criteria described above were highly effective in allowing the systematic removal of bot responses, such procedures only eliminated three-quarters of the problem, while the remaining quarter of fraudulent responses required time-consuming scrutiny and manual elimination through participant phone outreach. During phone dialing, we found that a high number of participant phone numbers led to inactive lines, while many others led to business lines where the participants of interest did not appear to work (as confirmed verbally). This suggests that some bots are creating random phone numbers while others are pulling numbers from online sources (e.g., company webpages). The interference of bots and need for a comprehensive identification of bot activity adversely affected our original goal of providing a timely summary of emerging assessment findings to residents.

Based on the prior literature, additional steps to help screen bot responses out of online survey data include the use of the Completely Automated Public Turing test to tell Computers and Humans Apart (CAPTCHA), which often requires users to type in letters and numbers from a distorted image that a computer cannot copy [67]. This tool, however, is not a feature of all survey software (as exemplified by its absence from this study) and may decrease participation of individuals with low computer literacy or visual impairment [68]. Another screening option is to require participants to first sign up for the study using an online form, after which a survey link is sent only to those participants (allowing for pre-vetting). Another strategy recommended by Teitcher et al. (2015) is the extraction of IP address, which can allow researchers to remove data from participants originating from outside the study area as well as repeated IP addresses (indicating that the same participant may have participated multiple times), each of which can indicate bot activity [68]. However, this step is not foolproof given that IP addresses can be faked, students may take a survey from the same computer, and that no official database exists that links IP addresses to locations (thus requiring researchers to rely on private companies).

While social media can be an effective means of survey circulation, platforms like Facebook and Twitter are also considered ideal spaces for bot hackers seeking research studies. In our experience, major influxes of bot responses indeed occur immediately following the public dissemination of survey links on Facebook. For that reason, avoiding the public circulation of survey links on social media is likely a useful step in minimizing bot attacks [69]. However, findings from this study indicate that, in an environmental crisis, affected communities rely on social media to exchange information and resources, thus making social media an appropriate tool for recruiting participants for a needs assessment. Weighing these tradeoffs is therefore an important consideration. It has been noted that bots can “learn,” and will improve upon their responses the longer they have access to a survey, thus making bot detection more difficult [67]. In general, it is advisable to closely monitor incoming data when a survey link is published and to shut down the link and create a new one if bot activity is suspected (as bots may return to the same survey link numerous times).

Though not applicable in this study, based on our prior work with online surveys, we have also found the identification of contradicting responses to be useful in identifying bot activity (e.g., a male selecting “yes” as to whether they are pregnant). In general, we found the use of automated bot screening procedures paired with a subsequent screening step of manual phone dialing of participants to be a feasible and highly effective way of removing fraudulent responses, the latter step allowing us to circumvent even the most sophisticated bots. Though potentially time- and resource-intensive, when paired with an automated pre-screening procedure, this can be readily feasible over a period of just a few hours, especially with multiple research assistants.

### 4.4. Strengths and Limitations of This Study

An important strength of this study is that it is grounded in community concerns and observations as well as principles of community-driven community–academic partnerships [70,71,72,73]. The geographic coverage, design, and implementation of this study, along with the ongoing development of a vision for a more informed and resilient community were each guided by our partnership process. Community–academic partnerships characterized by ownership of action research agendas by community and academic partners have greater potential for informing the translation of research into action to promote community health and health equity [70,73]. An additional strength of this study is its geographic relevance to exposure, encompassing only residents living within 10 miles of the fire.

Limitations of this study include the limited sample size and geographic focus, which reduces the generalizability of our findings to the wider county. Since the survey was disseminated online (and open to all local residents), we also could not ensure a targeted survey pool (e.g., those residing directly under plume paths or nearest the fire site). One way to overcome this in future surveys is to conduct door-to-door survey outreach (this would also reduce web-bot influence). Additionally, the voluntary nature of survey participation may have resulted in selection bias that resulted in an overestimate of health impacts. Relatedly, our assessments of mental and other health impacts were also restricted to cross-sectional analyses, and did not enable the casual attribution of such health impacts to the Tustin hangar fire directly. Similarly, the health outcomes in this study were all self-reported and cannot be confirmed to be clinically meaningful. Since the survey was disseminated more than two months after the cessation of the fire, participant recall of specific details may have also been impacted. The survey was also cross-sectional in nature and was therefore unable to account for opinions and perspectives that may have changed over time. Lastly, the survey was mostly successful in reaching the general resident population, whereas the teachers-only survey yielded very limited results. While the current study cannot assess long-term impacts of the fire incident on community health and resilience, such investigation may be worthwhile for future research.

## 5. Conclusions

This study presents an analysis of a survey disseminated to residents living near a major structure fire that burned for 24 days in California, most of whom were exposed to ash and smoke. Mental health outcomes were widely reported among residents, including anxiety, fatigue, headaches, stress, fear, and depression, as were physical health impacts, including respiratory and cardiovascular symptoms and nose/sinus irritation. Among those experiencing economic impacts such as missed work and money spent on remediation, lower-income residents were disproportionately represented. Health-protective actions were adopted by many residents, although critical knowledge gaps were identified, such as residents not using air purifiers despite owning them and/or using them improperly. Communities overwhelmingly reported disapproval about their city’s and/or government’s clarity and effectiveness in communicating fire-related information and health guidelines, although variability was observed by city. Findings from this study underscore the potential impacts of major pollution events on neighboring communities and offer critical insights to better position government agencies to respond during future disasters while effectively communicating with the public and addressing community needs.

## Figures and Tables

**Table 1:** Characteristics of sample population (N = 54).

Race/Ethnicity	N	%
American Indian/Alaska Native	2	3.7
Asian American/Asian	6	11.1
Black/African American	0	0.0
Middle Eastern/North African	2	3.7
Native Hawaiian/Other Pacific Islander	1	1.9
Latina/o/x/é	26	48.2
White/Caucasian	19	35.2
Other	2	3.7
Age		
18–24	18	33.3
25–34	21	38.9
35–44	12	22.2
45–59	3	5.6
≥60	0	0.0
Gender		
Male	22	40.74
Female	29	53.7
Non-Binary/Other	1	1.85
Education		
Junior College or below	9	16.7
Some College	8	14.8
Bachelor’s Degree or above	35	64.8
Annual Household Income		
<USD 25 K	3	5.6
USD 25–50 K	10	18.5
USD 50–75 K	13	24.1
USD 75–100 K	12	22.2
USD 100–200 K	7	13.0
≥USD 200 K	2	3.7

Note: not all percentages sum to 100% since participants had the option to select “prefer not to answer” and because one question (about race/ethnicity) used “check-all-that-apply” format.

## Data Availability

Data related to this study will be made available by the authors upon request.

## Supplementary Materials

The following supporting information can be downloaded at: https://www.mdpi.com/article/10.3390/ijerph22071003/s1, Figure S1: Conceptual framework guiding key research questions; Figure S2: Residents’ reporting of respiratory and cardiovascular symptoms during or immediately after the Tustin Hanger fire; Figure S3: Residents’ reporting of skin- and eye-related symptoms during or immediately after the Tustin Hanger fire.
