# Supplementary material for: A Community-Based Assessment of Attitudes, Health Impacts and Protective Actions During the 24-Day Hangar Fire in Tustin, California
Source: Int J Environ Res Public Health. 2025 Jun 26;22(7):1003. doi: 10.3390/ijerph22071003 (PMC12295795; doi:10.3390/ijerph22071003)
Supplement: Supplementary file 1 [file ijerph-22-01003-s001.zip › ijerph-3620695-supplementary.pdf]

Supplemental Materials

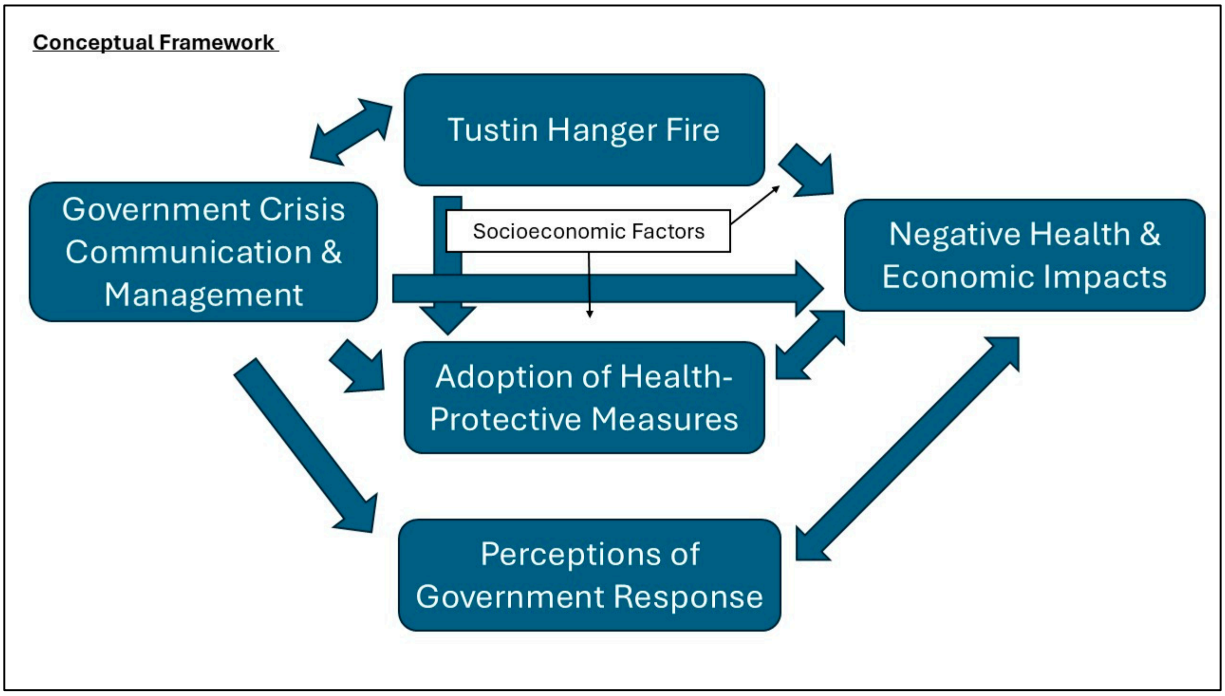

Figure S1. Conceptual framework guiding key research questions.

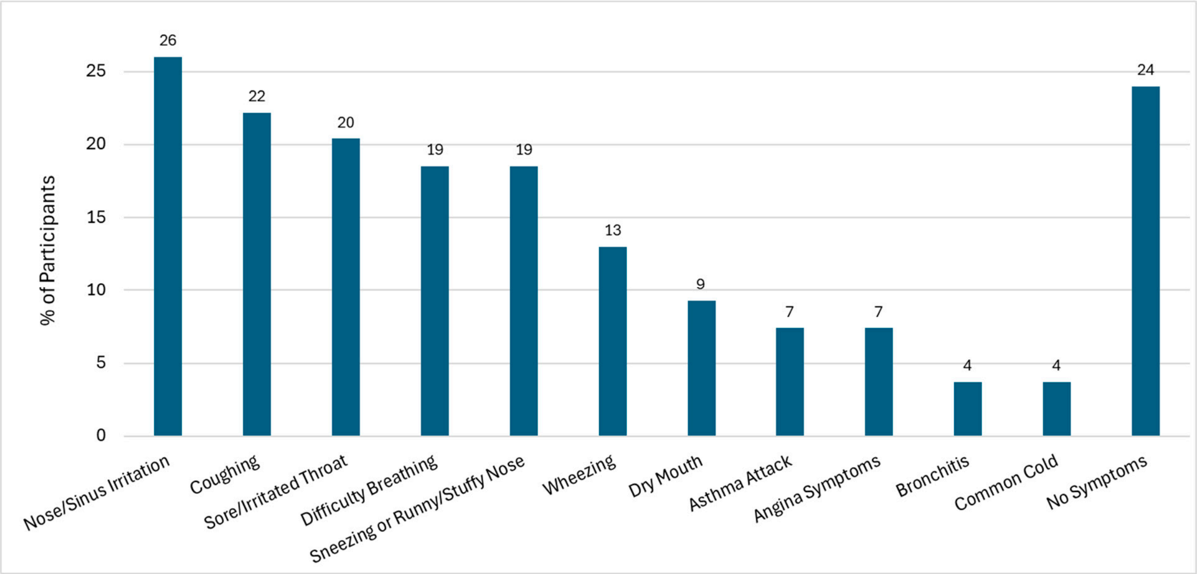

Figure S2. Residents' reporting of respiratory and cardiovascular symptoms during or immediately after the Tustin Hanger fire.

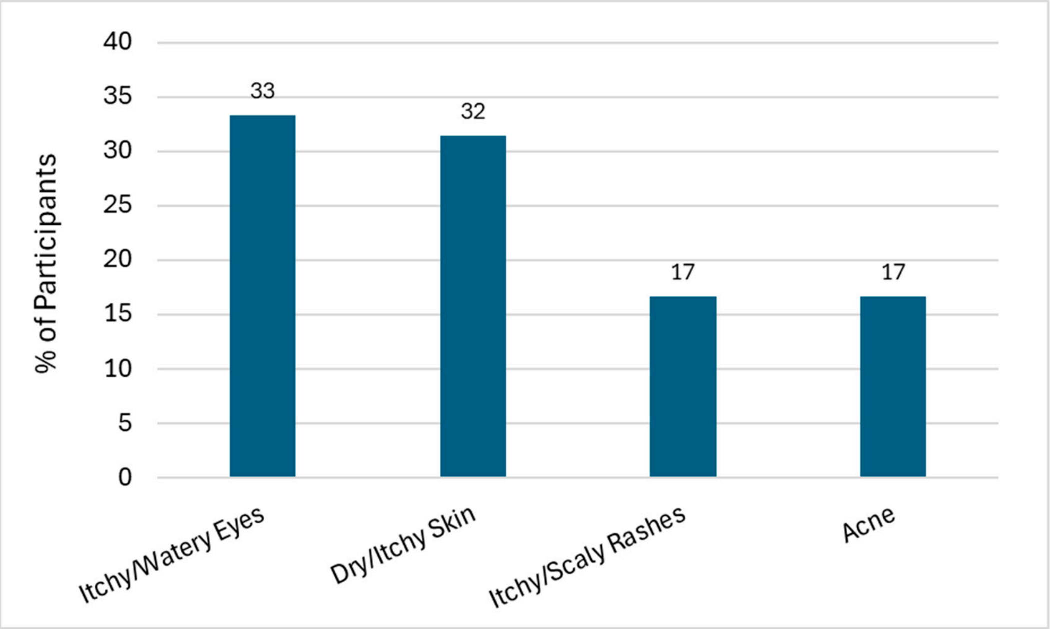

Figure S3. Residents' reporting of skin- and eye-related symptoms during or immediately after the Tustin Hanger fire.
